# The Silencing of a 14-3-3ɛ Homolog in *Tenebrio molitor* Leads to Increased Antimicrobial Activity in Hemocyte and Reduces Larval Survivability

**DOI:** 10.3390/genes7080053

**Published:** 2016-08-20

**Authors:** Gi Won Seo, Yong Hun Jo, Jeong Hwan Seong, Ki Beom Park, Bharat Bhusan Patnaik, Hamisi Tindwa, Sun-Am Kim, Yong Seok Lee, Yu Jung Kim, Yeon Soo Han

**Affiliations:** 1 Division of Plant Biotechnology, Institute of Environmentally-Friendly Agriculture (IEFA), College of Agriculture and Life Sciences, Chonnam National University, Gwangju 500-757, Korea; ndnd2@nate.com (G.W.S.); yhun1228@jnu.ac.kr (Y.H.J.); dmdtkal222@nate.com (J.H.S.); misson112@naver.com (K.B.P.); 2 Trident School of Biotech Sciences, Trident Academy of Creative Technology, Chandrasekharpur, Bhubaneswar 751024, India; drbharatbhusan4@gmail.com; 3 The Soil Microbiology Laboratory, Department of Soil and Geological Sciences, College of Agriculture, Sokoine University of Agriculture, P.O. Box 3008, Chuo Kikuu, Morogoro, Tanzania; tindwa@yahoo.com; 4 Jeonnam Bioindustry Foundation Bio Control Research Center, Gokseong 516-942, Korea; sunam0907@naver.com; 5 Department of Life Science and Biotechnology, College of Natural Sciences, Soonchunhyang University, Asan 336-745, Korea; yslee@sch.ac.kr; 6 Department of Chemistry and Biochemistry, College of Natural Sciences, California State University, San Bernardino, CA 92407, USA; yjkim@csusb.edu

**Keywords:** *Tenebrio molitor*, Tm14-3-3ɛ, AMP secretion, RNA interference, innate immunity

## Abstract

The 14-3-3 family of phosphorylated serine-binding proteins acts as signaling molecules in biological processes such as metabolism, division, differentiation, autophagy, and apoptosis. Herein, we report the requirement of 14-3-3ɛ isoform from *Tenebrio molitor* (Tm14-3-3ɛ) in the hemocyte antimicrobial activity. The *Tm14-3-3ɛ* transcript is 771 nucleotides in length and encodes a polypeptide of 256 amino acid residues. The protein has the typical 14-3-3 domain, the nuclear export signal (NES) sequence, and the peptide binding residues. The *Tm14-3-3ɛ* transcript shows a significant three-fold expression in the hemocyte of *T. molitor* larvae when infected with *Escherichia coli* *Tm14-3-3ɛ* silenced larvae show significantly lower survival rates when infected with *E. coli*. Under *Tm14-3-3ɛ* silenced condition, a strong antimicrobial activity is elicited in the hemocyte of the host inoculated with *E. coli*. This suggests impaired secretion of antimicrobial peptides (AMP) into the hemolymph. Furthermore, a reduction in AMP secretion under *Tm14-3-3ɛ* silenced condition would be responsible for loss in the capacity to kill bacteria and might explain the reduced survivability of the larvae upon *E. coli* challenge. This shows that Tm14-3-3ɛ is required to maintain innate immunity in *T. molitor* by enabling antimicrobial secretion into the hemolymph and explains the functional specialization of the isoform.

## 1. Introduction

The 14-3-3 family constitutes a highly conserved group of proteins present ubiquitously in all eukaryotic organisms. These proteins exist as multiple isoforms in eukaryotic cells which may explain their functional redundancy. In mammals, there are at least seven 14-3-3 isoforms (β, Ɛ, γ, η, ζ, θ and σ), named after their order of elution from reverse-phase high-performance liquid chromatography [1,2]. These isoforms form homodimeric and heterodimeric complexes that provide an interaction surface for phosphorylated-serine target proteins [2]. The individual isoforms are largely identical across species with few regions of diversity that may explain the evolution of the 14-3-3 family before the separation of insects, plants, amphibians, and mammals into separate lineages. The 14-3-3 isoforms are variously coined as “G-box Factor 14-3-3” (GF14) in higher plants [3], brain modulosignalin homolog (BMH) in yeast [4], and protein kinase-C inhibitor (KCIP) in sheep [5]. Interestingly, in insect models, only two 14-3-3 homologs, 14-3-3ɛ and 14-3-3ζ, have been reported. The *Drosophila* 14-3-3ɛ (Dm14-3-3ɛ) and 14-3-3ζ (Dm14-3-3*ζ*) isoforms show a high sequence identity of 82% and 88% to their respective mammalian orthologues [6,7]. Homologs of the *14-3-3ɛ* and *14-3-3ζ* gene are also characterized from lepidopteron models such as *Plutella xylostella* [8] and *Spodoptera litura* [9], respectively. The silkworm 14-3-3 proteins (Bm14-3-3ɛ and Bm14-3-3ζ) show a sequence identity of almost 85%–90% with *Drosophila* 14-3-3 isoforms [10]. These suggest functional conservation with diversity of binding attributed to the hyper-variable C-terminal segment of 14-3-3 proteins [11,12]. The 14-3-3 proteins essentially function in the regulation of cell proliferation, differentiation, metabolism, and apoptosis by binding to diverse signaling proteins, including kinases, phosphatases, and transmembrane receptors [13,14].

The Dm14-3-3ɛ isoform shows partial redundancy with 14-3-3ζ isoform for RAS1 signaling in photoreceptor formation and animal viability [15]. The Dm14-3-3ɛ is also responsible for controlling growth and apoptosis [16]. Recently, the Dm14-3-3ɛ isoform was implicated in innate immunity by regulating the secretion of antimicrobial peptides from hemocyte to hemolymph in response to bacterial infection [7,17]. In contrast, Dm14-3-3ζ was known to be involved in phagocytosis [18]. In addition to *Drosophila*, 14-3-3ɛ and ζ isoforms have been cloned from other insects including *Bombyx mori*. These isoforms were shown to bind to Hsp60 in larval and adult tissues of *B. mori* and were suggested to work together with Hsp60 to achieve broad cellular functions [19].

The mealworm beetle, *Tenebrio molitor* is the most elegant and efficient model for an explorative study on the innate immune mechanisms in beetles. Several studies, including ours, are focused towards delineating the mechanisms of the Toll [20,21,22,23,24,25,26,27,28], prophenoloxidase cascade [29,30] and autophagy signaling pathways [31,32,33] in *T. molitor* with special reference to immunity against microorganisms. We have also taken advantage of the *T. molitor* model to show the requirement of apolipophorin-III in conferring immunity against an intracellular Gram-positive bacteria, *Listeria monocytogenes* [34,35]. However, the understanding of antimicrobial peptide (AMP) secretion from hemocyte to hemolymph and the consequent bactericidal action is mostly unknown in this model insect. To gain further insights into the same, we cloned and characterized the *Tm14-3-3ɛ* from *T. molitor*. RNA interference (RNAi) knockdown techniques demonstrated that Tm14-3-3ɛ may play an important role in antimicrobial action in the hemocyte, affecting the secretion of AMPs to the hemolymph, and consequently increasing the larval mortality against *Escherichia coli* infection.

## 2. Materials and Methods

### 2.1. Insect Rearing, Microorganism Culture, and Challenge Experiments

*T. molitor* larvae were reared on an artificial diet (4.4 g of NeoVita, 0.5 g of chloramphenicol, 0.4 g of l-ascorbic acid, 0.5 g of sorbic acid, 0.5 mL of propionic acid, 2.2 g of yeast dry powder, 2.2 g of bean powder, 7.6 g of agar, 4.4 g of wheat powder and 73.3 g of wheat bran in 200 mL of distilled water; autoclaved at 121 °C for 15 min) in an insectary at 26 ± 1 °C, 60% ± 5% relative humidity and at dark condition.

*Escherichia coli* strain K12 and *Staphylococcus aureus* strain RN4220 were obtained from Pusan National University, Pusan, Korea and the mother culture of *Candida albicans* was procured from Hoseo University, Asan, Korea. *E. coli* and *S. aureus* were cultured in Luria-Bertani (LB) broth and *C. albicans* was cultured in Sabouraud dextrose broth overnight at 37 °C. The cells were harvested, washed twice in phosphate-buffered saline (PBS), and were centrifuged at 3500 rpm for 10 min. The cultured cells were diluted with PBS to reach a concentration of 10^9^ cells/mL of *E. coli* and *S. aureus,* and 5 × 10^7^ cells/mL of *C. albicans*.

During challenge experiments, 12th instar *T. molitor* larvae were microinjected with 1 µL suspensions of *E. coli* (10^6^ cells/µL), *S. aureus* (10^6^ cells/µL), or *C. albicans* (5 × 10^4^ cells/µL) using Picospritzer III micro-dispense system (Parker, Hollis, NH, USA). The control larvae were injected with 1 µL of PBS at the same time.

### 2.2. Full-Length cDNA Cloning and in Silico Analysis of the Putative Protein

Partial cDNA sequence of *Tm14-3-3*ɛ was screened from *T. molitor* expressed sequence tag (EST) and RNAseq database. The full-length cDNA sequence of *Tm14-3-3*ɛ was obtained by 5’- and 3’-Rapid amplification of cDNA ends-polymerase chain reaction (RACE-PCR). The cDNA templates for RACE-PCR were prepared using SMARTer^TM^ RACE cDNA amplification kit (Clontech laboratories, Mountain View, CA, USA) according to manufacturer’s instructions. The gene-specific primers, nested primers, and the RACE-PCR SMART universal primers were used for amplification (Table 1). The 5’- and 3’-end RACE amplification was conducted using the program: 30 cycles of denaturation at 94 °C for 30 s, annealing at 55 °C for 30 s, and extension at 72 °C for 30 s. The PCR products were purified, cloned into TOPO TA cloning vector (Invitrogen Corporation, Carlsbad, CA, USA), and subsequently transformed into competent *E. coli* DH5α cells. The transformants were screened by colony PCR and sequenced.

The specific domains in the deduced amino acid sequence of Tm14-3-3ɛ were analyzed by InterProScan (http://www.ebi.ac.uk/Tools/pfa/iprscan/) and BLAST (http://blast.ncbi.nlm.nih.gov/) programs. A phylogenetic tree based on the maximum-likelihood method was constructed by compiling the amino acid sequences of Tm14-3-3ɛorthologs from GenBank and analysis using ClustalX2 [36] and MEGA6 [37] programs.

### 2.3. Expression Analysis of Tm14-3-3ɛ Transcripts and RNAi

To identify the gene expression patterns of *Tm14-3-3ɛ* transcripts in fat body, gut, and hemocyte tissues of *E. coli*-infected and PBS-injected (control) larval groups, samples were collected 12 h post-infection, and total RNA isolated using FavorPrep™ Tri-RNA Reagent (Favorgen biotech Corp., Ping-Tung, Taiwan). The cDNAs were synthesized from total RNA (1 µg) with AccuPower® RT Pre Mix (Bioneer Co., Daejeon, Korea) and Oligo (dT)_12–18_ primer using a PTC-200 real-time PCR system (Bio-Rad, Hercules, CA, USA). The quantitative real-time PCR (qRT-PCR) was conducted using Exicycler-96 real-time system (Bioneer Co., Daejeon, Korea) under the reaction conditions as follows: initial denaturation at 95 °C for 20 s followed by 40 cycles at 95 °C for 5 s, and 60 °C for 20 s. The gene-specific primers for qRT-PCR were designed with Primer 3 software and listed in Table 1. The *T. molitor* housekeeping gene, *60S ribosomal protein L27a* (*TmL27a*) was used as an internal reference. Data from three independent observations were recorded and represented as the mean ± SE (*n* = 3).

For RNA interference analysis, the primers (Table 1) for *Tm14-3-3ɛ* and *Tm14-3-3ζ* were designed using the SnapDragon dsRNA design software (http://www.flyrnai.org/cgi-bin/RNAi_find_primers.pl) to minimize off-target effects. Subsequent to PCR amplification, the purified PCR products were used for dsRNA synthesis using the Ampliscribe T7-flash transcription kit (Epicentre Biotechnologies, Madison, WI, USA). The dsRNA for *Tm14-3-3ɛ* (5 µg per larva) and *Tm14-3-3ζ* (1 µg per larva) were injected into last instar *T. molitor* larva. RNAi efficiency was observed for a period of 5 days to confirm the target gene silencing. *Enhanced green fluorescent protein* (*EGFP*) dsRNA synthesized from pEGFP-C1 plasmid vector (Clontech Laboratories, Mountain View, CA, USA) was used as the negative control.

### 2.4. Larval Mortality Assay

After the confirmation of *Tm14-3-3ɛ* gene silencing, the dsRNA injected groups (ds*Tm14-3-3ɛ* and ds*EGFP*) were inoculated with *E. coli* suspension (10^6^ cells per larva), and the larval mortality was monitored for a period of seven days. The study was conducted with 50 larvae, and the results represent an average of three biological replications. The cumulative survival rates were considered significant (*p* < 0.05) after conducting the Wilcoxon Mann Whitney test. 

### 2.5. Antibacterial Activity Assay

Antibacterial activity of the hemolymph and hemocyte lysate against *E. coli* was assayed using the CFU count method [38]. For the same, *E. coli* (10^7^ cells per larva) were injected into ds*Tm14-3-3ɛ* and ds*Tm14-3-3ζ* treated larva, and allowed incubation within the insect larva for 12h. PBS and *E. coli* only (10^7^ cells per larva) injected groups acted as negative and positive controls, respectively. The hemolymph from the larvae (pooled) was collected by cutting the proleg with sterile scissors and collected in a tube containing decoagulation buffer (DECO buffer; 15 mM sodium chloride, 30 mM trisodium citrate, 26 mM citric acid, 20 mM EDTA, pH 5.0). The hemolymph was centrifuged at 3500 rpm for 10 min at 4 °C to sediment the hemocytes. Hemocyte and hemolymph samples were boiled at 100 °C for 10 min, and centrifuged at 15,000 rpm for 10 min at 4 °C. The optical density at OD_220_ of the supernatant was measured to estimate total protein. Bactericidal activity of hemolymph and hemocyte lysate was monitored using *E. coli* as an indicator bacterium. For the activity assay, the collected hemolymph and hemocyte lysate were diluted serially with PBS, and a portion (50 μg of peptides) of diluted samples was incubated with 10^6^ *E. coli* cells in 200 μL of insect saline (130 mM NaCl, 5 Mm KCl, 1 mM CaCl_2_) for 2 h at 37 °C. Subsequently, the mixture was diluted 2000-fold with insect saline and 100 μL aliquots were spread on LB agar plates. The plates were incubated for 16 h at 37 °C and the colony numbers on test and control plates were compared.

### 2.6. Statistical Analysis

All data are shown as means ± S.E. The difference between group means is assessed by one-way analysis of variance (ANOVA) and Tukey’s multiple range tests at 95% confidence level (*p* < 0.05) using SAS 9.1.3 for Windows (SAS Institute, Cary, NC, USA).

## 3. Results and Discussion

### 3.1. Molecular Cloning and Sequence Analysis of Tm14-3-3ɛ

**Figure 1:**
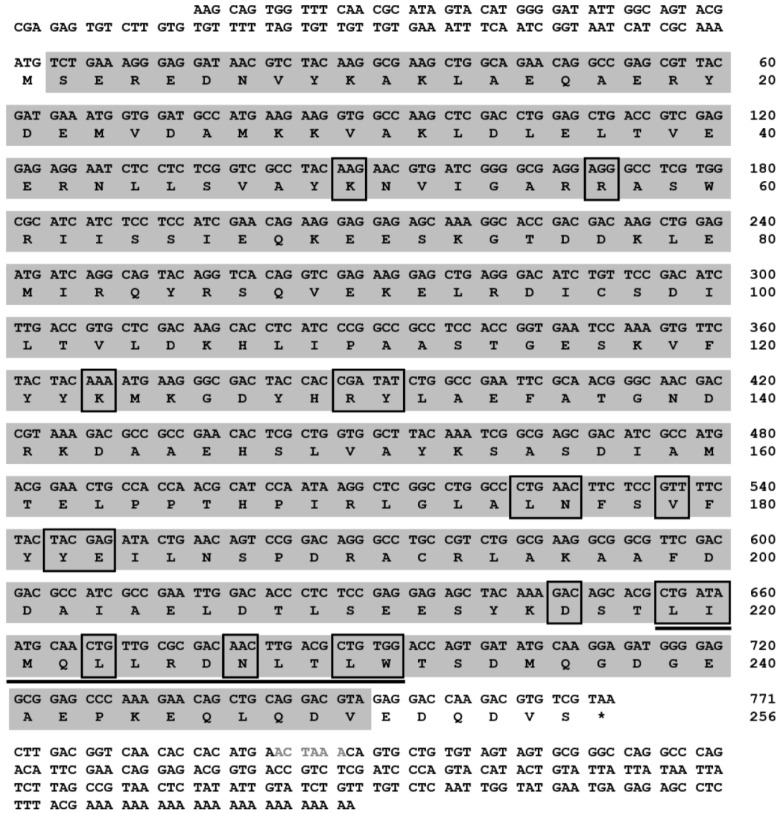
Tm14-3-3ε cDNA and deduced amino acid sequence information. The nucleotides are numbered from the first base of the translation start codon (ATG). The polyadenylation signal sequence in 3’-UTR is shown in grey text. The conserved 14-3-3 domain is shown in the grey shaded box. The direct peptide binding residues are boxed. The nuclear export sequence (NES) is shown underlined.

We first retrieved a partial coding sequence of the *14-3-3ɛ* gene from *Tenebrio* RNA-seq and EST database. After confirmation and validation of the full-length cDNA sequence of *Tm14-3-3ɛ* gene by cloning and sequencing, the information was submitted in GenBank under the accession number KP099937. The ORF of *Tm14-3-3ɛ* gene is composed of 771 nucleotides and encodes a protein of 256 amino acid residues. The *Tm14-3-3ɛ* gene shows a 5’-untranslated region (UTR) and a 3’-UTR of 105 bp and 209 bp, respectively. Furthermore, a polyadenylation signal (ACTAAA) was identified 22 nucleotides downstream of the stop codon. The deduced Tm14-3-3ɛ protein includes a conserved middle core region, called the 14-3-3 domain that participates as the main functional domain for interaction with partner proteins. In addition, a nuclear export signal (NES) sequence of 13 amino acids (N-LIMQLLRDNLTLW-C) has been identified in the 14-3-3ɛ protein of *T. molitor* that includes the presence of a few phosphopeptide-binding residues (Leu-219, Ile-220, Leu-223, Asn-227, Leu-230, and Trp-231). The NES signal sequence is found highly conserved in most of the identified 14-3-3 proteins and is critical for shuttling partner proteins in the nucleus [39]. The nucleotide and deduced amino acid sequences of Tm14-3-3ɛ with the domain information are shown in Figure 1.

**Figure 2:**
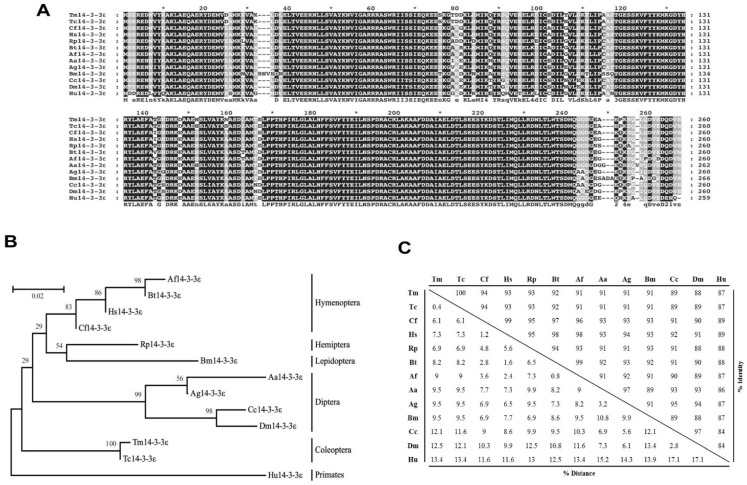
Multiple alignment, phylogenetic analysis, and percentage identity of 14-3-3ε. (**A**) Multiple alignment was conducted by Clustal X2 program and visualized by GeneDoc software; (**B**) Molecular phylogenetics of Tm14-3-3Ɛ with other 14-3-3Ɛ isoforms from insects. *Homo sapiens* 14-3-3Ɛ sequence was used as outgroup. A bootstrap consensus tree is shown based on the Jones-Taylor-Thornton matrix model; (**C**) Percentage identity and distance were analyzed by Clustal X2 and MEGA6 program. The GenBank accession numbers of the analyzed sequences are as follows: Tm14-3-3ε; *T. molitor* 14-3-3 protein epsilon, Tc14-3-3ε; *T. castaneum* 14-3-3 protein epsilon (XP_969719.1), Cf14-3-3ε; *Camponotus floridanus* 14-3-3 protein epsilon (EFN68394.1), Af14-3-3ε; *Apis florea* 14-3-3 protein epsilon-like (XP_003698936.1), Bt14-3-3ε; *Bombus terrestris*14-3-3 protein epsilon-like (XP_003397232.1), Rp14-3-3ε; *Riptortus pedestris*14-3-3 protein epsilon (BAN20616.1), Hs14-3-3ε; *Harpegnathos saltator*14-3-3 protein epsilon (EFN89235.1), Bm14-3-3ε; *Bombyx mori*14-3-3 protein epsilon (NP_001091764.1), Aa14-3-3ε; *Aedes aegypti*14-3-3 protein epsilon (XP_001655109.1), Ag14-3-3ε; *Anopheles gambiae* str. PEST 14-3-3 protein epsilon (XP_322009.2), Cc14-3-3ε; *Ceratitis capitata* 14-3-3 protein epsilon-like (XP_004537104.1), Dm14-3-3ε; *Drosophila melanogaster* 14-3-3 protein epsilon, isoform C (NP_732312.1), Hu14-3-3ε; *Homo sapiens* 14-3-3 protein epsilon (NP_006752.1).

A multiple sequence alignment analysis of Tm14-3-3ɛ isoform was conducted with 14-3-3ɛ proteins from insect orders to determine the evolutionary relationships and conservation within the critical 14-3-3 domain (Figure 2A). The evolutionary relationship of Tm14-3-3ɛ amino acid sequence with 14-3-3ɛ proteins from other insects shows well-spaced clusters. The 14-3-3ɛ proteins from the beetle partners, *T. molitor* and *Tribolium castaneum* were branched as separate cluster with 100% identity. The percentage identity analysis also shows a high (91%–94%) proximity to other insect 14-3-3ɛ proteins. Remarkably, *Tenebrio* 14-3-3ɛ shows a high (88%) identity to Dm14-3-3ɛ (Dm14-3-3ɛ) suggesting functional redundancy of the protein isoform ”ɛ” among the insects. Although this investigation did not include the analysis of the isoform-specific differences, it does show the putative occurrence of functional redundancy among the insect 14-3-3ɛ proteins. In any case, it was reported that the 14-3-3ɛ proteins from invertebrates group show domain sequence similarity with the non-epsilon isoforms from mammals highlighting their functional similarity with the ancestral protein [40]. The phylogenetic tree and the percent identity analysis of the 14-3-3ɛ protein from *T. molitor* have been shown in Figure 2B,C, respectively.

### 3.2. Expression of Tm14-3-3ɛ Transcripts and Innate Immune Function in Hosts

The *Tenebrio14-3-3ɛ* transcript shows a consistent expression throughout development and was ubiquitously detected in all the larval and adult tissues. This finding is consistent to the previous report of *Dm14-3-3ɛ* expressed during development and also in almost all embryonic and larval tissues [7]. The *P. xylostella* 14-3-3ɛ (Px14-3-3ɛ) protein also shows a constant expression in various tissue types and during the developmental stages of the moth [8]. The broad expression patterns of 14-3-3ɛ proteins can be reflected in their regulatory function in modulating many cellular processes [12,41].

**Figure 3:**
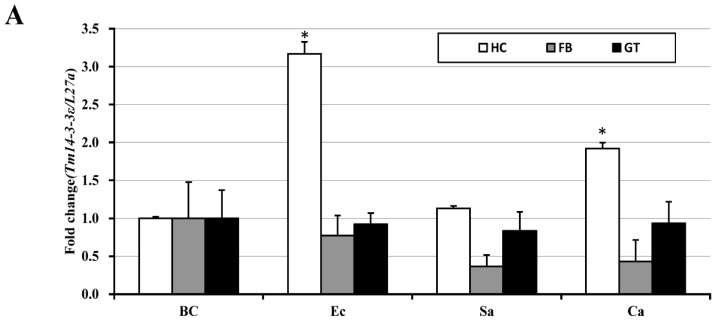

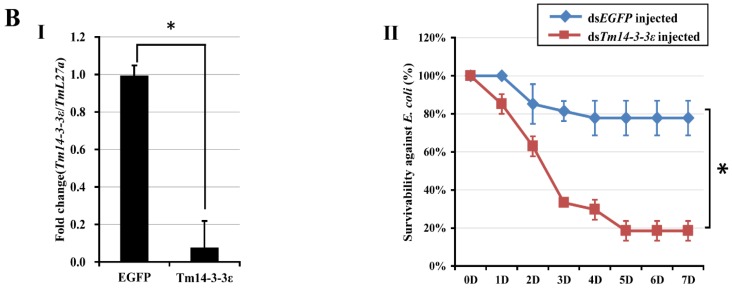
Expression analysis and RNA interference-based silencing of *Tm14-3-3ε* transcripts in *T. molitor.* (**A**) A qPCR analysis show the expression of *Tm14-3-3ε* transcripts in fat body, gut, and hemocytes of the host after the inoculation of *Escherichia coli* (Ec), *Staphylococcus aureus* (Sa), and *Candida albicans* (Ca). PBS injected larval group act as injection control. Total RNA was extracted from the tissues 12 h post-injection and profiled by qPCR. The housekeeping gene, *TmL27a* acts as an endogenous control. The experiments are conducted in three biological replications (*n* = 3). Bars represent mean ± standard error. * *p* < 0.05 (SAS, ANOVA); (**B**) (**I**). Silencing efficiency of *Tm14-3-3ε* transcripts in the whole-body of *T. molitor* larva after six days of dsRNA injection; (**II**). Time-dependent survival of ds*EGFP* and ds*Tm14-3-3ε* injected *T. molitor* larval groups after the inoculation of *E. coli*. For each group, at least *n* = 100 individual larvae were analyzed and results are shown as mean ± S.E. The percent mortality was recorded for at least seven days, and the experiment was conducted in three biological replications. Statistical significance was conferred by Wilcoxon-Mann-Whitney test (*p* < 0.05).

To study the putative function of *Tenebrio* 14-3-3ɛ protein in innate immunity, we studied the expression levels of the transcript in fat body, gut, and hemocyte after challenge with microorganisms such as *E. coli*, *S. aureus*, and *C. albicans* (Figure 3A). A significant rise in the *Tm14-3-3ɛ* mRNA levels was noticed in the hemocyte (nearly three-fold expression) in the case of *E. coli* infection. A small but significant rise in *Tm14-3-3ɛ* transcript level in the hemocyte was also noticed in the case of *C. albicans* infection. The increase in expression of *Tm14-3-3ɛ* mRNA by *E. coli* (Gram-negative bacteria) and *C. albicans* (fungus) and not by *S. aureus* (Gram-positive bacteria) in this study could be related to the specific interaction with cell surface pathogen receptors. As demonstrated before in *Tenebrio* and *Drosophila*, fungal and Gram-positive bacteria associated molecular patterns such as β-glucan and Lys-type peptidoglycan are recognized by Gram-negative binding protein 3 (GNBP-3) and PGRP-SA-GNBP-1, respectively. But unlike *Drosophila*, the monomeric DAP-type peptidoglycan (Gram-negative bacteria associated molecular patterns) induces the release of diptericin-like antimicrobial peptide in *Tenebrio* hemocytes [25]. We suspect Tm14-3-*3ɛ* acts as an immune regulator and its expression on *E. coli* infection would influence the release of *Drosophila* diptericin-like antimicrobial peptides. Furthermore, it relates to the hemocyte-specific functions of *Tm14-3-3ɛ* transcripts. The expression level of the *Tm14-3-3ɛ* transcripts was measured after 12 h of inoculation into the host and statistically compared to the buffer-injected control. The *Tm14-3-3ɛ* mRNA expression analysis data provided leads to enquire about the hemocyte immune response in the *E. coli* infected larvae of the host. To date, only the *Drosophila* model system has been studied in detail regarding the role of 14-3-3ɛ in innate immunity [7]. Considering an 88% sequence similarity between Tm14-3-3ɛ and Dm14-3-3ɛ proteins that suggests functional conservation, we hypothesized the requirement of Tm14-3-3ɛ in conferring immunity to the host against *E. coli* infection. For the same, we generated specific dsRNA to silence the *Tm14-3-3ɛ* transcripts in the host. We observed about 95% silencing (significant at the level of *p* < 0.05) of the transcript in the ds*Tm14-3-3ɛ* injected group, when compared with the ds*EGFP* injected group (Figure 3B). Under this condition, we infected either the ds*EGFP* or *Tm14-3-3ɛ* silenced *Tenebrio* larvae, through the systemic route. The 12th instar larvae were challenged with a suspension of Gram-negative bacteria, *E. coli*. The surviving larvae were scored every day for the entire duration of the assay (for seven days, Figure 3C). We observed that the survival of the ds*EGFP* injected group was not significantly affected by *E. coli* infection (81% by seven days), but under *Tm14-3-3ɛ* silenced condition the survival of *T. molitor* larvae was dramatically reduced (18% by 7 days) when compared with the ds*EGFP* injected control group. This shows that *Tm14-3-3ɛ* silenced *Tenebrio* larvae are susceptible to death following Gram-negative bacterial challenge and that Tm14-3-3ɛ is necessary to ward off a possible infection by the pathogen in the host. The high mortality of *Tenebrio* larvae in *Tm14-3-3ɛ* silenced condition upon *E. coli* infection suggests a possible relationship of the *Tm14-3-3ɛ* transcript with immune system defects in the host. This has been elucidated succinctly in *Dm14-3-3ɛ* mutants that show decreased survival in response to Gram-negative *E. coli* and Gram-positive *Micrococcus luteus* infection when compared with wild-type strains [7].

### 3.3. Tm14-3-3ɛ and Hemocyte Antimicrobial Activity

**Figure 4:**
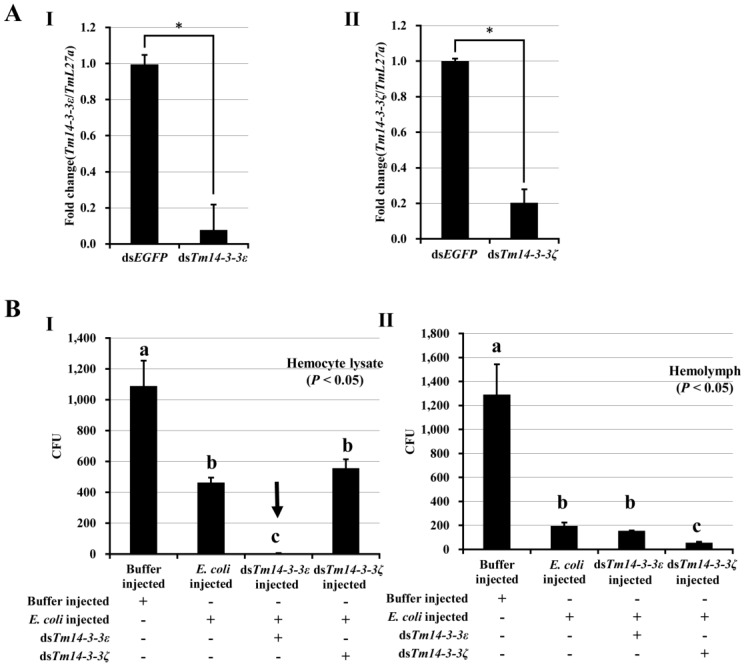
Antimicrobial function of *Tm14-3-3ɛ* transcripts in *T. molitor.* (**A**) RNA interference and antimicrobial activity study of *Tm14-3-3ε* and *Tm14-3-3ζ*. Knockdown efficiency of *Tm14-3-3ε* (I) and *Tm14-3-3ζ* (II) transcripts in the whole-body of *T. molitor* larva six days after dsRNA injection. ds*EGFP* was used as a negative control. The data represent the mean ± S.E. of three independent biological replications; (**B**) Antimicrobial activity assay in hemocyte (**I**) and hemolymph (**II**) of *Tm14-3-3ε* and *Tm14-3-3ζ* silenced groups. Buffer injected and only *E. coli* injected groups is used as negative control and positive control, respectively. Bars represent mean ± standard error of three independent biological replications. One-way ANOVA analysis of variances should significant differences between group means (*p* < 0.05). Different subscripts over bars depict significant differences between means of groups.

A high *14-3-3ɛ* transcript level in the hemocyte of *Tenebrio* larvae and the reduced survival of *Tm14-3-3ɛ* silenced larvae to infection by *E. coli* suggests a role for the transcript in the activation of the hemocyte antimicrobial activity. This is consistent with previous reports showing the regulatory function of *Dm14-3-3ɛ* in the secretion of AMPs from hemocytes to hemolymph [7,17]. To determine whether *Tm14-3-3ɛ* plays a similar role, we made a successful attempt in silencing the *Tm14-3-3ɛ* (93% silencing; *p* < 0.05; Figure 4A-I) and *Tm14-3-3ζ* (80% silencing; *p* < 0.05; Figure 4A-II) transcripts in two separate RNAi studies. *Tm14-3-3ζ* gene characterized in a separate study showed high degree of conservation at the amino acid sequence level with Tm14-3-3ε and ε isoforms from other insects. The phylogenetic analysis located 14-3-3ε and ζ isoforms of *T. molitor* to separate clusters of ε and ζ sequences (Supplementary Figure S1). RNAi knockdown of the *14-3-3ζ* isoform in *T. molitor* was performed to examine its possible role in the antimicrobial response of hemocytes and hemolymph to Gram-negative *E. coli* infections. We had three groups of *E. coli* inoculation, including the *Tm14-3-3ɛ* and *Tm14-3-3ζ* silenced larval groups. A buffer injected group without *E. coli* injection was used as the negative control. We could find a significant increase (*p* < 0.05) of antimicrobial activity in hemocyte of *Tm14-3-3ɛ* silenced/*E. coli* injected group (lane 3; Figure 4B-I), in comparison with *Tm14-3-3ζ* silenced/*E. coli* injected group and *E. coli* only injected group (lane 2; Figure 4B-I). In the hemolymph, a significantly high (*p* < 0.05) reduction in colony-forming units (cfu) was noticed in *Tm14-3-3ζ* silenced/*E. coli* group (Lane 4; Figure 4B-II). This suggests that silencing of *Tm14-3-3ɛ* and not *Tm14-3-3ζ* transcripts significantly increase the antimicrobial activity in the hemocyte of the infected host, thereby putatively leading to a decreased secretion of AMPs from hemocyte into the hemolymph. We suspect the drop in secretion of AMP into the hemolymph would significantly affect the bactericidal action in the hemolymph. This potentially explains the multiplication of *E. coli* in the *Tenebrio* larvae and an increased mortality in *Tm14-3-3ɛ* silenced condition. The putative role of Tm14-3-3ε as an adaptor protein regulating the exocytosis of AMPs has been reported for Dm14-3-3ε through elegant studies. *Drosophila* mutant for 14-3-3ε show decreased survivability against bacterial infections due to the loss of exocytosis functions [17]. From the present study, we also conclude that *Tm14-3-3ζ* does not show hemocyte antimicrobial function, suggesting the normal secretion of the AMPs and consequent bactericidal action. We could assume that the silencing of *Tm14-3-3ζ* could be implicated in the phagocytosis of microbes and would impede the survival of the larvae after *E. coli* infection. In an earlier report, *Dm14-3-3ζ* silencing had been attributed to compromised larval survival rate due to the phagocytosis of *S. aureus* [18]. Herein, we argue that the loss of *14-3-3ɛ* in *Tenebrio* larvae may putatively lead to reduced AMP in the hemolymph due to enhanced antimicrobial action in the hemocyte. A regulatory loss/reduction in AMP secretion may result in increased mortality when infected with *E. coli*.

## 4. Conclusions

In this study, we show that the 14-3-3ɛ isoform cloned and characterized from the model insect *T*. *molitor* is required to control host viability under bacterial challenge. This crucial function in *Tm14-3-3ɛ* silenced larvae is possibly due to a reduction in AMP secretion from hemocyte to the hemolymph. Lately, we have observed that the *Tm14-3-3ɛ* silenced larvae show a decreased survival in response to the Gram-positive bacterial and fungal challenge. We would be interested in providing necessary insights for the high mortality rate and the role of Tm14-3-3ɛ to elicit a common innate immune mechanism against the pathogens.

## Figures and Tables

**Table 1:** Primer sequences used in the present study.

Name	Primer sequences
Tm14-3-3ε5’-RACE GSP1 Tm14-3-3ε 5’-RACE nGSP2	5’-GCTCCTTCTCGACCTGTGAC-3’ 5’-ACCTTCTTCATGGCATCCAC-3’
Tm14-3-3ε 3’-RACE GSP1 Tm14-3-3ε 3’-RACE nGSP2	5’-CTCGCTGGTGGCTTACAAAT-3’ 5’-GCAGGACGTAGAGGACCAAG-3’
dsTm14-3-3ε-Fw dsTm14-3-3ε-Rv	5’-TAATACGACTCACTATAGGGAGAACAGGTCGAGAAGGAGCTGA-3’ 5’-TAATACGACTCACTATAGGGAGAACGTCCTGCAGCTGTTCTTT-3’
dsTm14-3-3ζ-Fw dsTm14-3-3ζ-Rv	5’-TAATACGACTCACTATAGGGTAGAAACGGGCGTAGAACTCA-3’ 5’-TAATACGACTCACTATAGGGTGCATCATCGAAAGCCTGTTT-3’
dsEGFP-Fw dsEGFP-Rv	5’-TAATACGACTCACTATAGGGTACGTAAACGGCCACAAGTTC-3’ 5’-TAATACGACTCACTATAGGGTTGCTCAGGTAGTGGTTGTCG-3’
Tm14-3-3ε-qPCR-Fw Tm14-3-3ε-qPCR-Rv	5’-TGGTGGATGCCATGAAGAAG-3’ 5’-CTGTTCGATGGAGGAGATGATG-3’
Tm14-3-3ζ-qPCR-Fw Tm14-3-3ζ-qPCR-Rv	5’-TTTGGCGGAAGTAGCCACAGGAGA-3’ 5’-TAATCTGATGGGATGTGTGGGCGT-3’
TmL27a-qPCR-Fw TmL27a-qPCR-Rv	5’-TCATCCTGAAGGCAAAGCTCCAGT-3’ 5’-AGGTTGGTTAGGCAGGCACCTTTA-3’

Polymerase recognition signals have been underlined.

## Supplementary Materials

Supplementary Figure S1 is available online at www.mdpi.com/2073-4425/7/8/53/s1.
