# Supplementary material for: The Silencing of a 14-3-3ɛ Homolog in Tenebrio molitor Leads to Increased Antimicrobial Activity in Hemocyte and Reduces Larval Survivability
Source: Genes (Basel). 2016 Aug 20;7(8):53. doi: 10.3390/genes7080053 (PMC4999841; doi:10.3390/genes7080053)
Supplement: Supplementary file 1 [file genes-07-00053-s001.pdf]

The Silencing of a 14-3-3ε Homolog in *Tenebrio molitor* Leads to Increased Antimicrobial Activity in Hemocyte and Reduces Larval Survivability

Gi Won Seo [1,†], Yong Hun Jo [1,†], Jeong Hwan Seong [1], Ki Beom Park [1], Bharat Bhusan Patnaik [2], Hamisi Tindwa [3], Sun-Am Kim [4], Yong Seok Lee [5], Yu Jung Kim [6] and Yeon Soo Han [1,*]

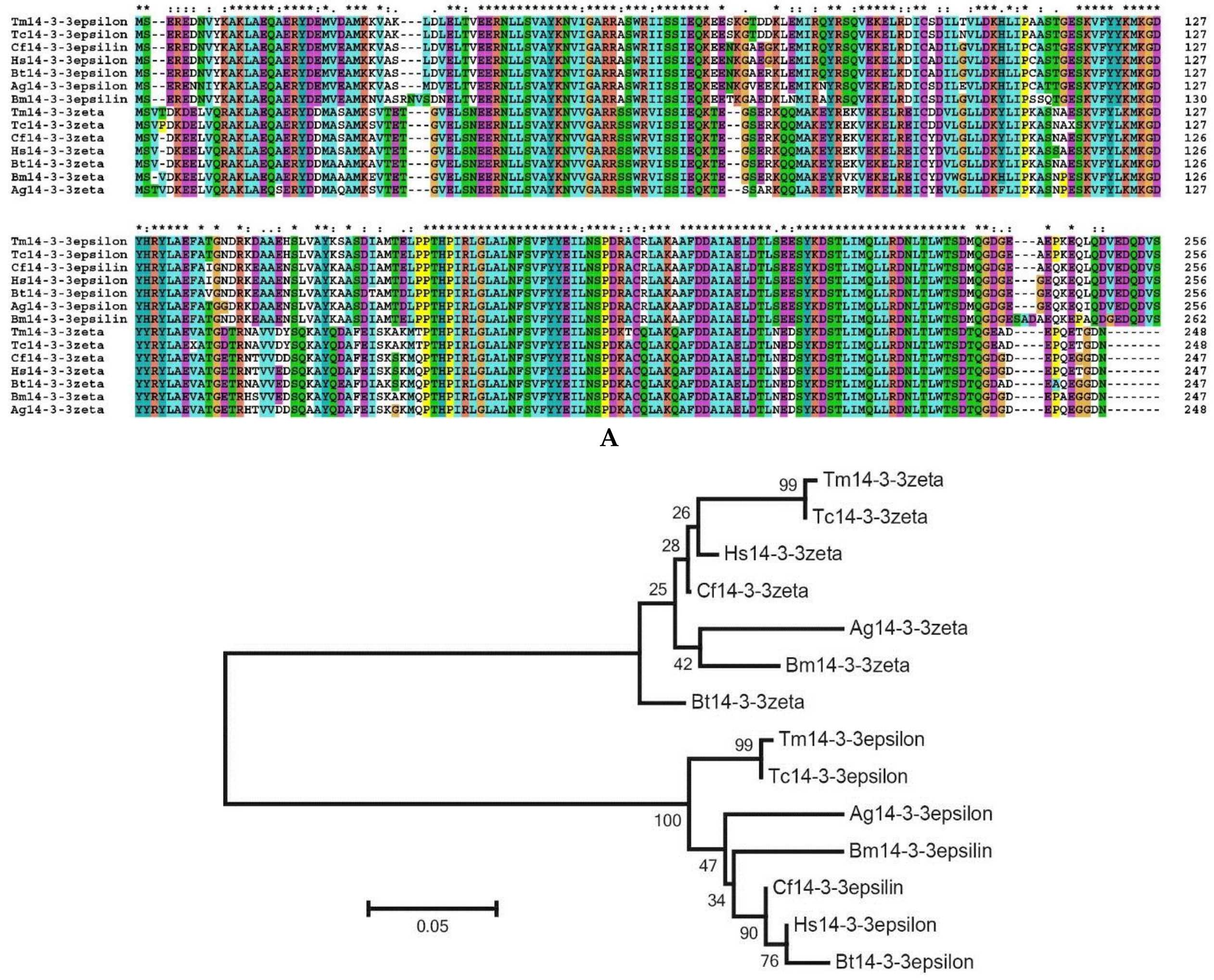

Figure S1. Multiple alignment and phylogenetic analysis of Tenebrio 14-3-3 epsilon and zeta with other 14-3-3 epsilon and zeta from insects. (**A**) Amino acid sequences of insects 14-3-3 proteins were obtained from GenBank and multiple alignment of 14-3-3 proteins was performed with Clustal X2 program. The result shows that the insect 14-3-3 proteins were highly conserved. The C-terminus region of 14-3-3 epsilon and zeta shows the specific amino acid sequences; (**B**) Phylogenetic analysis of 14-3-3 proteins were conducted with MEGA 6.2 program and the result indicates that the 14-3-3 epsilon and 14-3-3 zeta proteins were located on the separate branches. Tm14-3-3epsilon; *T. molitor* 14-3-3 epsilon, Tm14-3-3zeta; *T. molitor* 14-3-3 zeta, Tc14-3-3epsilon; *Tribolium castaneum* 14-3-3 protein epsilon (XP_969719.1), Tc14-3-3zeta; *T. castaneum* 14-3-3 protein zeta (XP_008200725.1), Cf14-3-3epsilin; *Camponotus floridanus* 14-3-3 protein epsilon (EFN68394.1), Cf14-3-3zeta; *C. floridanus* 14-3-3 zeta (AEW70330.1), Hs14-3-3 epsilon; *Harpegnathos saltator* 14-3-3 protein epsilon (EFN89235.1), Hs14-3-3zeta; *H. saltator* 14-3-3 zeta (AEW70352.1), Bt14-3-3epsilon; *Bombus terrestris* 14-3-3 protein epsilon-like (XP_003397232.1), Bt14-3-3zeta; *B. terrestris* 14-3-3 zeta (AEW70333.1), Ag14-3-3epsilon; *Anopheles gambiae* str. PEST 14-3-3 epsilon (XP_322009.2), Ag14-3-3zeta; *A. gambiae* str. PEST 14-3-3 epsilon 2L:48608644-48613035 (AGAP007643-PB), Bm14-3-3epsilin; *Bombyx mori* 14-3-3 epsilon protein (NP_001091764.1), Bm14-3-3zeta; *B. mori* 14-3-3 protein zeta (NP_001040164.1).
